# Giving birth in Brazilian Birthing Centers: care outcomes from Freestanding Birth Centers

**DOI:** 10.11606/s1518-8787.2026060007624

**Published:** 2026-10-26

**Authors:** Gisele Almeida Lopes, Nathalie Leister, Cláudia de Azevedo Aguiar, Jamile Claro de Castro Bussadori, Bruna Dias Alonso, Maria Luiza Riesco

**Affiliations:** I Universidade de São Paulo Escola de Enfermagem Departamento de Enfermagem Materno-Infantil e Psiquiátrica São Paulo SP Brasil Universidade de São Paulo. Escola de Enfermagem. Departamento de Enfermagem Materno-Infantil e Psiquiátrica. São Paulo, SP, Brasil; II Universidade Federal do Triângulo Mineiro Instituto de Ciências da Saúde Departamento de Saúde Coletiva Uberaba MG Brasil Universidade Federal do Triângulo Mineiro. Instituto de Ciências da Saúde. Departamento de Saúde Coletiva. Uberaba, MG, Brasil; III Universidade Federal de São Carlos Departamento de Enfermagem São Carlos SP Brasil Universidade Federal de São Carlos. Departamento de Enfermagem. São Carlos, SP, Brasil; IV Centro Universitário das Américas São Paulo SP Brasil Centro Universitário das Américas – FAM. São Paulo, SP, Brasil

**Keywords:** Birthing Centers, Natural Childbirth, Midwifery, Midwives

## Abstract

**OBJECTIVE:**

To analyze care outcomes related to monitoring and evaluation indicators at Freestanding Birth Centers operating in Brazil.

**METHODS:**

A nationwide cross-sectional study integrated with the Nascer no Brasil II initiative. The study included the eight Freestanding Birth Centers that attended to more than 100 births in 2019; the sample comprised all women admitted to these centers, provided they met the inclusion criteria. The outcomes corresponded to the 12 monitoring indicators for Freestanding Birth Centers defined by the Ministry of Health, including data on care, interventions, transfers, and maternal and neonatal outcomes.

**RESULTS:**

The vaginal birth rate was 91.8%, while the cesarean section rate was6.2%. The rates of episiotomy (2.0%), amniotomy (14.5%), and oxytocin use (12.7%) were low. Most women had six or more prenatal visits (94.5%) and were accompanied by a support person during hospitalization (97.7%). Newborns with an Apgar score of less than seven at five minutes of life accounted for 0.4%, and 93.3% had immediate and uninterrupted skin-to-skin contact. The intrapartum transfer rate was 19.3%, the postpartum transfer rate was 1.5%, and the neonatal transfer rate was 2.9%.

**CONCLUSIONS:**

The Freestanding Birth Centers showed low rates of interventions and care based on best practices, resulting in favorable maternal and neonatal outcomes, indicating a safe, evidence-based care model for low-risk women.

## INTRODUCTION

Most pregnant women experience the pregnancy and postpartum period without major complications^1^. For this reason, for years, the World Health Organization (WHO) has published recommendations based on scientific evidence for a positive maternal experience during prenatal care, childbirth, and the postpartum period, including safe and respectful care^2^. At the same time, the WHO has endorsed robust studies indicating that there are no justifications or maternal-perinatal benefits for cesarean section rates exceeding 10% to 15%^6,7^.

Freestanding Birth Centers (FBC) are health facilities designed to provide care for low-risk births, located near hospitals to ensure rapid access to medical and technological resources in the event of a transfer. Regulated by Ministry of Health (MoH), Ordinance No. 11/2015 FBC aim to provide a welcoming and safe environment where pregnant women can experience childbirth in a humanized manner, with interventions used judiciously^8^. These centers are led by midwives and nurse-midwives -professionals trained to attend to normal, low-risk births without the need for medical professionals to be present. This model aligns with WHO recommendations, which recognize that these health professionals can safely manage labor, especially in low-risk settings^4^.

The creation and expansion of FBC in Brazil are part of a broader strategy to reduce the excessive medicalization of childbirth and address the alarming rates of cesarean sections. According to data from the MoH^9^, the cesarean section rate in the country was 60.5% in 2024, and according to the Agência Nacional de Saúde Suplementar (National Supplementary Health Agency), in the private sector this rate is approximately 80%^10^. These figures are well above the WHO recommendation, which states that rates between 10% and 15% would be sufficient to ensure maternal and neonatal safety. Cesarean sections without clinical indication are associated with a higher risk of obstetric and neonatal complications, including infections, hemorrhage, and neonatal respiratory distress^6,11^.

On the other hand, the FBC care model, by encouraging spontaneous vaginal birth, respecting women’s choices, and promoting a humanized care environment, directly contributes to reducing these complications, offering safe care based on scientific evidence^12^. However, despite the favorable evidence and efforts to implement and expand this care model, in 2024, 98% of births in the country occurred in hospitals, which highlights the limited incorporation of FBC into childbirth care in Brazil^9^.

The implementation of the FBC in Brazil is linked to the Rede Cegonha (Stork Network), recently renamed the Rede Alyne (Alyne Network), as part of a strategy to restructure the maternal and child health care model in Brazil. This thematic and priority network of the Unified Health System (SUS) aims to guarantee women the right to reproductive planning, humane care during pregnancy, childbirth, and the postpartum period, and to ensure children’s right to a safe birth and healthy development^16^.

In addition to the national guidelines^17^, the care provided at FBC in Brazil is aligned with the global maternal health goals established by the WHO. The organization maintains that the care model at FBC, which is woman-centered and led by nurse-midwives and midwives, has the potential to reduce maternal and neonatal morbidity and mortality in low-risk settings^4^. International studies support this approach, demonstrating that births taking place in low-risk care facilities have outcomes that are equally safe-or even better-when compared to those in conventional hospital settings, provided there is adequate infrastructure and strict criteria for the admission of pregnant women^14,15^. This approach is reinforced by evidence showing that births attended by midwives and nurse-midwives are associated with lower rates of interventions, such as cesarean sections, episiotomies, and the use of oxytocin, in addition to promoting greater maternal satisfaction and better neonatal outcomes^13^.

Despite the favorable evidence, FBC remain limited in their integration into childbirth care in Brazil, and local scientific literature still lacks studies with national scope that evaluate their outcomes based on standardized indicators. Given this scenario, this study aims to analyze care outcomes related to monitoring and evaluation indicators of FBC operating within the country’s SUS.

## METHODS

### Study Type

Birth in Brazilian Birthing Centers is a cross-sectional, descriptive, and analytical study of national scope, integrated into Nascer no Brasil II. The protocol detailing the study methodology is available in Leister et al.^18^

### Study Site

The Birth in Brazilian Birthing Centers study includes all eight FBC facilities in the country that recorded more than 100 births in 2019 in the Sistema de Informações sobre Nascidos Vivos (Live Birth Information System), as well as their referral hospitals for the collection of data on intrapartum, postpartum, and neonatal transfer outcomes. At the time of FBC selection, there were 13 FBCs listed in the Cadastro Nacional de Estabelecimentos de Saúde (National Registry of Health Facilities); however, only eight were eligible for the study. The facilities are located in São Paulo (n = 2), Bahia (n = 1), the Federal District (n = 1), Minas Gerais (n = 1), Pará (n = 1), Pernambuco (n = 1), and Rio de Janeiro (n = 1). Data on the selection of FBC centers and hospitals for the study are available in Leister et al.^18^

### Participants in the Data Collection Study

The study population consisted of all pregnant women admitted for labor at one of the FBC facilities included in the study. Pregnant women who left the birthing center or hospital before discharge, who were transferred or had their baby transferred to a hospital other than the FBC’s referral hospital, who legally relinquished custody of their baby, and those who did not understand Portuguese were not invited to participate.

Data collection took place from September 2022 to March 2025 by a team of approximately 40 people, all of whom were nurses or midwives, through interviews and medical record data. During the study period, 1,631 women were admitted to the eight FBC. A total of 1,407 women were included in the sample (Chart).

**Chart:** Number of births in 2019, planned and collected sample in “Nascer nas Casas de Parto,” Brazil, 2022–2025.

Birthing center	Births in 2019	Planned sample	Collected sample
Birthing center 1	886	468	471
Birthing center 2	639	337	177^a^
Birthing center 3	408	215	215
Birthing center 4	407	215	216
Birthing center 5	211	113	115
Birthing center 6	153	81	81
Birthing center 7	130	69	70
Birthing center 8	118	62	62
**TOTAL**	**2,952**	**1,560**	**1,407**

^a^ Closure of the Freestanding Birth Centre during the data collection period.

### Monitoring and Evaluation Indicators for Freestanding Birth Centres (FBC)

The monitoring and evaluation indicators for FBC were analyzed, as established in MoH Ordinance, which regulates the operation and monitoring of these services within the SUS. These indicators include parameters related to the admission of pregnant women, management of labor, interventions performed, maternal and neonatal outcomes, as well as intrapartum, postpartum, and neonatal transfer rates^8^.

### Study Variables

#### Exposure: Sociodemographic and Obstetric Characteristics

The variables were: Age in years (continuous, calculated based on date of birth); Race/ethnicity (White; Black; Asian; Brown; Indigenous); Educational attainment (incomplete elementary school; complete elementary school; complete high school; complete college or higher); Paid employment (no, yes); Marital status (no partner; has a partner); Number of pregnancies (continuous); Number of previous vaginal births (continuous); Number of previous cesarean sections (continuous); Gestational age at birth - continuous (in weeks); Risk factors in the current pregnancy (descriptive).

## Outcome

These correspond to all 12 FBC monitoring and evaluation indicators, which were divided into:

**a.Data on births**

Indicator 1:

1a) number of births attended;

1b) percentage of births attended by midwifery and medical professionals;

1c) percentage of adolescent births;

1d) Percentage of water births;

1e) Percentage of births in an upright position.

**b.Data on the perineum**

Indicator 2: Percentage of episiotomies.

Indicator 3: Percentage of first- and second-degree tears.

Indicator 4: Percentage of third- and fourth-degree tears

**c.Data on interventions**

Indicator 5: Percentage of amniotomy .

Indicator 6: Percentage of births with oxytocin in the second stage of labor.

**d.Data on companions**

Indicator 7: Percentage of women with a support person during labor, birth, and the postpartum period.

**e.Data on prenatal care**

Indicator 8: Percentage of women who received six or more prenatal visits.

**f.Data on the newborn (NB):**

Indicator 9:

9a) Percentage of NB with a birth weight less than 2,500 g or greater than 4,000 g;

9b) Percentage of NB with a gestational age of less than 37 weeks and of more than 41 weeks;

9c) Percentage of NB with an Apgar score of less than seven at the fifth minute of life;

9d) Percentage of NB with immediate and uninterrupted skin-to-skin contact after birth.

**g.Data on transfers**

Indicator 10: Percentage of intrapartum transfers, broken down by reason for transfer.

Indicator 11:

11a) Percentage of postpartum transfers, broken down by reason for transfer;

11b) Percentage of neonatal transfers, broken down by reason for transfer.

**h.Data on Medical Care**

Indicator 12: Percentage of obstetric or pediatric medical evaluations/procedures at the FBC (refers to the proportion of evaluations or procedures performed by an obstetrician or pediatrician on the FBC’s own premises).

**i.Other relevant data: type of birth and Kristeller maneuver.**

## Data Analysis

The data were analyzed using the statistical software R (R Foundation for Statistical Computing) in a descriptive manner, with absolute and relative values for each outcome.

## Ethical Considerations

The study was approved by the Research Ethics Committees of the Escola Nacional de Saúde Pública Sergio Arouca of the Fundação Oswaldo Cruz (opinion no. 5.192.349); the Fundação de Ensino e Pesquisa em Ciências da Saúde (opinion no. 5.243.029); Hospital Sofia Feldman/Fundação de Assistência Integral à Saúde (opinion no. 5.256.617); the Rio de Janeiro Municipal Health Secretariat (opinion no. 5.218.319); and the São Paulo Municipal Health Secretariat (opinion no 5.251.421). Upon being invited to participate in the study, the participants signed the Informed Consent Form, authorizing the interview and the collection of data from their institutional records.

## RESULTS

During the period from September 2022 to March 2025, 1,631 women were admitted to the FBC, 62 (3.8%) did not meet the eligibility criteria. Among the 1,569 eligible women, 52 (3.3%) refused to participate in the study. Additionally, there were 110 (7.0%) losses due to the postpartum women being discharged before recruitment efforts began.

The socioeconomic and obstetric characteristics are presented in Table 1. The participants were 26.5 years old on average, and slightly more than half self-identified as Brown (52.4%). Most had completed high school (57.0%), were in a relationship (87.8%), and were employed (55.5%). Regarding obstetric data, the majority were primiparous, 44.2% had a previous vaginal birth, and only 2.4% had a previous cesarean section (neither of these figures is shown in the table). The vast majority gave birth at term (96.9%).

**Table 1:** Socioeconomic and obstetric characteristics of participants in the “Birth in Brazilian Birthing Centers” study. São Paulo, 2022–2024.

Variable	n or mean (SD)	% or maximum-minimum
Age (n = 1,407)	26.5 (5.8)	13–46
10 to 19 years	147	10.4
20 to 34 years	1,117	79.4
≥ 35 years	143	10.2
Race/ethnicity (n = 1,407)		
White	358	25.4
Black	295	21
Asian	8	0.6
Brown	737	52.4
Indigenous	9	0.6
Education level (n = 1,405)		
Incomplete elementary school	94	6.7
Complete elementary school	202	14.4
Complete high school	801	57.0
Complete higher education	308	21.9
Paid work (n = 1,406)		
Yes	781	55.5
No	625	44.5
Marital status (n = 1,405)		
In a relationship	1,233	87.8
Not in a relationship	172	12.2
Pregnancies (n = 1,405)	1.9 (1.1)	1–8
1	782	55.6
2 to 3 (1 to 2 previous births)	556	39.6
4 to 5 (≥ 3)	67	4.8
Previous vaginal births (n = 722)	1.3 (0.9)	0–7
None	101	14.0
1	412	57.1
2	147	20.4
3	40	5.5
≥ 4	22	3.0
Previous cesarean sections (n = 722)	0. 1 (0.2)	0–2
None	685	94.9
1	34	4.7
2	3	0.4
≥ 3	0	0.0
Gestational age at birth (n = 1,407)	39.0 (0.7)	36–41
< 37 weeks	1	0.1
37 to 40 weeks	1,324	96.9
41 weeks	65	4.7

SD: standard deviation.

Table 2 presents the results for indicators 1 through 6 and 12 of the MoH Ordinance. These relate to childbirth care, perineal outcomes, interventions, and medical care. All indicators refer to all births among women admitted for birth at the FBC facilities, regardless of where they gave birth-whether in birthing centers or at referral hospitals following intrapartum transfer. The rate of normal birth among the participants was 92.0%, followed by cesarean section (6.2%).

**Table 2:** Indicators 1 through 6 and 12 of Ministry of Health Ordinance No. 11/2015. Birth in Brazilian Birthing Centers, 2022–2025.

Indicator	n	%
Indicator 1a): number of births attended	1,407	100
Type of birth		
Normal	1,294	92.0
Cesarean section	87	6.2
Forceps/vacuum extractor	26	1.8
Indicator 1b): births attended by nursing and medical professionals (n = 1,406)^a^		
Nurse-midwife/midwife	1,202	85.5
Physician (obstetrician)	204	15.5
Indicator 1c): adolescent births (< 20 years)	147	10.4
Indicator 1d): water births^b^ (n = 1,294)	162	12.5
Indicator 1e): position for birth^b^ (n = 1,294)		
Dorsal lithotomy position	16	1.2
Lying on the back without leg supports	30	2.3
Non-lithotomy dorsal positions		
Sitting^c^	425	32.7
Semi-seated	374	28.8
All fours	176	13.6
Side	123	9.5
Squats^c^	103	8.0
Standing^c^	41	3.7
Other position	3	0.2
Indicator 2: episiotomy^b^ (n = 1,320)	29	2.2
Intact perineum	381	28.8
Tear, grade unspecified	27	2.0
Indicator 3: first- and second-degree tears^b^ (n = 1,320)	855	64.8
First-degree tear	457	34.6
Second-degree tear	398	30.2
Indicator 4: third- and fourth-degree tear^b^ (n = 1,320)	28	2.1
Third-degree tear	25	1.9
Fourth-degree tear	3	0.2
Indicator 5: amniotomy/ (n = 1,407)	310	22.0
Indicator 6: birth with oxytocin in the second stage (n = 1,407)	178	12.7
Kristeller maneuver (n = 1,407)	13	0.9

^a^ Only institutional records and interviews containing information about the healthcare professional who attended the birth were included.

^b^ Cesarean sections, forceps, and vacuum extractions were excluded.

^c^ Positions classified as vertical.

Most births were attended by nursing or midwifery professionals (85.5%), namely midwives, nurse-midwives, nurses without specialization, or midwifery residents. Adolescents accounted for 10.4% of the participants. The rate of water birth was 12.5%, and the sitting (32.7%) and semi-sitting (28.8%) positions were the most commonly adopted by the women in labor. An episiotomy was performed in 2.2% of the participants, and third- and fourth-degree tears occurred in 2.1% of them. Amniotomy was performed in 22.0% of births, and oxytocin was administered in 12.7%. The Kristeller maneuver, which is not included among the indicators but was added to Table 2, was performed in 0.9% of births.

Table 3 presents data related to indicators 7, 8, and 9 of MoH Ordinance No. 11/2015, which are: the presence of a support person throughout the hospital stay, the number of prenatal visits, and data on the babies’ births. From admission to discharge, 97.4% of the women had the uninterrupted presence of a support person. The lowest percentage of companion presence was during the analgesia procedure (81.5%). Most women had six or more prenatal visits (94.5%); 5.2% of the babies were born weighing less than 2,500 g or more than 4,000 g, and gestational ages of less than 37 weeks or more than 41 weeks accounted for 4.7% of births. Only 0.4% of newborns had an Apgar score of less than seven at five minutes of life, and the majority had uninterrupted skin-to-skin contact for one hour after birth (93.3%).

**Table 3:** . Indicators 7 through 9 of Ministry of Health Ordinance No. 11/2015. Birth in Brazilian Birthing Centers, 2022–2025.

Indicator	n	%
Indicator 7: presence of a support person during labor, birth, and the postpartum period (n = 1,390)	1,381^a^	97.4
Support person present at admission	1,386	99.7
Support person during labor	1,386	99.7
Support person during analgesia (n = 227^b^)	185	81.5
Support person during birth	1,382	99.4
Support person during the postpartum period	1,390	100
Indicator 8: prenatal care consisting of six or more prenatal visits (n = 1,368)	1,293	94.5
Indicator 9a): newborn weight < 2,500 g and > 4,000 g (n = 1,393)	73	5.2
Weight < 2,500 g	27	1.9
Weight > 4,000 g	46	3.3
Indicator 9b): gestational age of the newborn < 37 weeks and > 41 weeks (n = 1,390)	66	4.7
Gestational age < 37 weeks	1	0.1
Gestational age > 41 weeks	65	4.6
Indicator 9c): newborn Apgar score < 7 at 5 minutes (n = 1,401)	6	0.4
Indicator 9d): newborns with immediate and uninterrupted skin-to-skin contact after birth (n = 1,374)	1,282	93.3

^a^ The presence of a companion during analgesia was not considered in this calculation.

^b^ Number of births with analgesia.

Table 4 presents indicators 10 and 11 regarding intrapartum, postpartum maternal, and neonatal transfers, along with their respective reasons. The highest frequency of intrapartum transfers occurred for obstetric reasons, the main ones failure to progress (4.6%) and abnormal fetal heart rate (4.2%). Next, the laboring woman’s request for possible hospital-based analgesia stood out as a reason for transfer (4.2%). Regarding postpartum maternal transfers, clinical and obstetric reasons were prevalent, with the majority due to retained placenta (0.5%), hypertensive crisis (0.3%), and postpartum hemorrhage (0.3%). Among neonatal transfers, the main reasons were respiratory distress (0.9%) and early jaundice (0.8%).

**Table 4:** Indicators 10 through 11b of Ministry of Health Ordinance No. 11/2015. Birth in Brazilian Birthing Centers, 2022–2025.

Indicator	n	%
**Indicator 10: intrapartum transfer**	**272**	**19.3**
Obstetric reasons	190	13.5
Failure to progress (as defined by institutional protocol)	64	4.6
Abnormal fetal heart rate	59	4.2
Meconium	39	2.8
Prolonged rupture of membranes (as defined by institutional protocol)	26	1.8
Abnormal presentation	1	0.1
Tachysystole	1	0.1
Clinical causes	20	1.4
Hypertensive crisis or changes in vital signs	14	1.0
Abnormal laboratory test results	4	0.3
Asthma attack	1	0.1
Anxiety attack	1	0.1
Personal/professional/institutional reasons	62	4.4
Maternal request	59	4.2
On-call shifts without a clinician available	2	0.1
Contact with a person who tested positive for Covid-19	1	0.1
**Indicator 11a): postpartum transfer**	**21**	**1.5**
Obstetric reasons	11	0.9
Placental retention	6	0.5
Third- or fourth-degree perineal tear	4	0.3
Cervical laceration	1	0.1
Clinical causes	10	0.6
Hypertensive crisis	5	0.3
Postpartum hemorrhage	5	0.3
**Indicator 11b): neonatal transfer**	**41**	**2.9**
Obstetric reasons	1	0.1
Cephalohematoma	1	0.1
Clinical causes	39	2.8
Respiratory distress	13	0.9
Early jaundice	12	0.8
Heart rhythm abnormalities	4	0.3
Suspected infection	4	0.3
Suspected congenital abnormalities	3	0.2
Apnea	1	0.1
Hypoglycemia	1	0.1
Mother; positive VDRL^a^	1	0.1
Personal/professional/institutional reasons	1	0.1
Contact with a person who tested positive for Covid-19	1	0.1

^a^ VDRL (Venereal Disease Research Laboratory): a blood test used for syphilis screening and follow-up.

## DISCUSSION

This study describes care during labor and birth at eight Brazilian FBC facilities, based on indicators used by the MoH to monitor and evaluate these facilities. The data analysis shows that care at FBC facilities is of high quality for low-risk pregnancies, consistent with the findings of national and international studies.

Despite the favorable results, the operation and expansion of FBC facilities remain controversial issues. Of particular note are the limited availability and uneven distribution of these services nationwide, the need to strengthen integration with the hospital network, and institutional tensions related to the care model-factors that hinder their long-term consolidation and sustainability^8,12,19^. Nevertheless, in 2024, the federal government launched the new Programa de Aceleração do Crescimento (Novo PAC – Growth Acceleration Plan), which provides for investments in the construction and modernization of FBC facilities throughout the country^22^.

A crucial factor for the safety of care provided at FBC is the careful selection of women at low risk of complications. However, there is no global consensus on the precise definition of “low risk.” Some countries have guidelines for referral to a specialized professional during prenatal care, while others only have criteria for choosing the birth location. The existence of clear guidelines provides reassurance for both healthcare professionals and pregnant women, guiding referrals to safe birth facilities. In this regard, the Manual Técnico das Casas de Parto (Technical Manual for Freestanding Birth Centers) in the Municipality of São Paulo^23^stands out.

There remains a shortage of robust Brazilian studies evaluating maternal mortality and severe morbidity according to the place of birth, limiting the formulation of public policies based on consistent data. However, recent studies indicate growing interest in out-of-hospital care led by midwives and nurse-midwives, which yields neonatal outcomes similar to those in a hospital setting and is associated with lower rates of interventions such as episiotomies, cesarean sections, and the use of pharmacological pain relief^15,24,25^.

This study found high rates of normal birth (91.9%) among low-risk women who planned to give birth at a FBC, a result consistent with recent studies in Brazil and abroad^20,24^. The low rates of interventions, such as episiotomy, amniotomy, the use of oxytocin, and the Kristeller maneuver, combined with high rates of water immersion, the presence of a support person, and the use of non-lithotomy positions, indicate that the care provided is in line with WHO recommendations, prioritizing health promotion and a biopsychosocial and humanistic model of childbirth care^3,27,28^. These findings also suggest the incorporation of dimensions of safe and respectful care, such as continuous support and autonomy, broadening the concept of quality beyond biomedical outcomes^5^.

These results can be explained both by the profile of women who choose birth at FBC and by the model of care provided, which emphasizes individualized care (1:1) during labor. Meta-analyses by Bohren et al.^29^ and Sandall et al.^13^ demonstrate the benefits of the continuity-of-care model and individualized support, reflected in higher rates of spontaneous vaginal birth, lower use of interventions, instrumental births, and cesarean sections, without compromising neonatal outcomes, when compared to low-risk births in a hospital setting.

The frequencies of transfers during labor, postpartum, and the puerperium found in this study are similar to those reported in the international literature^24,26,30^. The stratification of reasons into obstetric, clinical, and institutional causes, a topic still under-explored in studies on FBC, broadens our understanding of the care dynamics of these services.

In the United States, the number of people choosing to give birth at FBC has doubled over the past 20 years. The U.S. experience highlights the importance of integrating FBC into the regional perinatal care system, as this improves outcomes by establishing defined referral pathways during prenatal care and childbirth, in addition to promoting effective communication and ongoing cooperation among professionals^31^.

Pregnant women must be guaranteed the right to information and informed choice, so that the decision regarding the location and mode of birth is made with full knowledge of the facts from the beginning of pregnancy, based on individual preferences and obstetric risk profile. It is essential that options regarding healthcare providers and settings be presented clearly and in a timely manner and that, for low-risk women, there is access to consistent evidence regarding outcomes in FBC^32,33^.

From this perspective, this study contributes by adopting an intention-to-treat analysis, taking into account the initial plan for birth in FBC, which allowed for the inclusion of all individuals admitted, regardless of transfer, thereby reducing selection bias and enhancing the internal validity of estimates of perinatal outcomes, including hospital-based outcomes. Furthermore, it provides data to support policies that encourage both births attended by midwives and nurse-midwives and births in out-of-hospital settings.

## CONCLUSIONS

In the FBC analyzed, low rates of interventions such as amniotomy, oxytocin use, the Kristeller maneuver, and lithotomy position at birth were observed, associated with high proportions of best practices, such as the presence of a support person, water birth, and uninterrupted skin-to-skin contact, constituting a care model aligned with contemporary recommendations for low-risk childbirth care. Among women with planned births in these services, the low rates of cesarean section (6.2%), instrumental vaginal births (1.8%), severe tears (2.1%), and Apgar scores lower than seven at the fifth minute of life (0.4%) reinforce the safety of the model, while transfers, predominantly motivated by non-emergency obstetric and clinical indications related to the need for monitoring and risk prevention, indicate adequate coordination with the hospital support system. Taken together, these results demonstrate the potential of FBC to improve perinatal care in Brazil by integrating clinical safety with the promotion of positive and respectful childbirth experiences.
