# Supplementary material for: Giving birth in Brazilian Birthing Centers: care outcomes from Freestanding Birth Centers
Source: Rev Saude Publica. 2026 Oct 26;60:e49. doi: 10.11606/s1518-8787.2026060007624 (PMC13753555; doi:10.11606/s1518-8787.2026060007624)
Supplement: Translation

# Translation: Nascer nas Casas de Parto do Brasil: resultados assistenciais de Centros de Parto Normal peri-hospitalares

**Authors:** Gisele Almeida Lopes, Nathalie Leister, Cláudia de Azevedo Aguiar, Jamile Claro de Castro Bussadori, Bruna Dias Alonso, Maria Luiza Riesco

**Affiliations:** I Brasil Universidade de São Paulo. Escola de Enfermagem. Departamento de Enfermagem Materno-Infantil e Psiquiátrica. São Paulo, SP, Brasil; II Brasil Universidade Federal do Triângulo Mineiro. Instituto de Ciências da Saúde. Departamento de Saúde Coletiva. Uberaba, MG, Brasil; III Brasil Universidade Federal de São Carlos. Departamento de Enfermagem. São Carlos, SP, Brasil; IV Brasil Centro Universitário das Américas – FAM. São Paulo, SP, Brasil

**Keywords:** Centros de Assistência à Gravidez e ao Parto, Parto Normal, Assistência ao Parto, Enfermagem Obstétrica

## Abstract

**OBJETIVO:**

Analisar os resultados assistenciais relacionados aos indicadores de monitoramento e avaliação dos Centros de Parto Normal peri-hospitalares em funcionamento no Brasil.

**MÉTODOS:**

Estudo transversal, de abrangência nacional, integrado ao Nascer no Brasil II. Foram incluídos os oito Centros de Parto Normal peri-hospitalares que prestaram assistência a mais de 100 partos no ano de 2019, e compuseram a amostra todas as mulheres admitidas nestes centros, desde que atendessem os critérios de inclusão. Os desfechos corresponderam aos 12 indicadores de monitoramento dos Centros de Parto Normal peri-hospitalares definidos pelo Ministério da Saúde, incluindo dados sobre a assistência, intervenções, transferências, desfechos maternos e neonatais.

**RESULTADOS:**

A taxa de parto vaginal foi de 91,8%, enquanto a de cesariana foi de 6,2%. As taxas de episiotomia (2,0%), amniotomia (14,5%) e uso de ocitocina (12,7%) foram baixas. A maioria das mulheres realizou seis ou mais consultas de pré-natal (94,5%) e contou com acompanhante durante a internação (97,7%). Recém-nascidos com Apgar menor que sete no quinto minuto de vida representaram 0,4%, e 93,3% tiveram contato pele a pele imediato e ininterrupto. A taxa de transferência intraparto foi de 19,3%, a pós-parto de 1,5% e a neonatal de 2,9%.

**CONCLUSÕES:**

Os Centros de Parto Normal peri-hospitalares apresentaram baixas taxas de intervenções e assistência baseada em boas práticas, resultando em desfechos maternos e neonatais favoráveis, indicando um modelo assistencial seguro e baseado em evidências para mulheres de baixo risco.

## INTRODUÇÃO

A maior parte das pessoas que engravidam vivencia o ciclo gravídico-puerperal sem grandes intercorrências^1^. Por esta razão, há anos, a Organização Mundial da Saúde (OMS) tem publicado recomendações baseadas em evidências científicas para uma experiência materna positiva no pré-natal, parto e puerpério, incluindo o cuidado seguro e respeitoso^2^. Em concomitância, a OMS tem ratificado estudos sólidos que indicam não haver justificativas e benefícios materno-perinatais para taxas de cesariana superiores a 10 % a 15%^6,7^.

Os Centros de Parto Normal peri-hospitalares (CPNp) são unidades de saúde destinadas ao atendimento de partos de baixo risco, localizadas próximas a hospitais, de modo a garantir acesso rápido a recursos médicos e tecnológicos em caso de necessidade de transferência. Regulamentados pela Portaria nº 11/2015 do Ministério da Saúde (MS), os CPNp têm como objetivo proporcionar um ambiente acolhedor e seguro, onde as gestantes possam vivenciar o parto de forma humanizada, com o uso de intervenções de forma criteriosa^8^. Esses centros são liderados por obstetrizes e enfermeiras obstétricas, profissionais capacitados para atender partos normais de baixo risco, sem a necessidade da presença de profissionais médicos. Essa configuração é alinhada às recomendações da OMS, que reconhece a condução do trabalho de parto com segurança por estas profissionais de saúde, especialmente em cenários de baixo risco^4^.

A criação e a expansão dos CPNp no Brasil fazem parte de uma estratégia mais ampla para reduzir a medicalização excessiva do parto e enfrentar o cenário de cesarianas em níveis alarmantes. Segundo dados do MS^9^, a taxa de cesarianas no país foi de 60,5% em 2024 e, de acordo com a Agência Nacional de Saúde Suplementar, no setor privado esse percentual é de aproximadamente 80%^10^. Trata-se de patamares muito acima da recomendação da OMS, que estabelece que taxas entre 10% e 15% seriam suficientes para garantir segurança materna e neonatal. As cesarianas sem indicação clínica estão associadas a maior risco de complicações obstétricas e neonatais, incluindo infecções, hemorragias e dificuldades respiratórias neonatais^6,11^.

Por outro lado, o modelo assistencial dos CPNp, ao incentivar o parto vaginal espontâneo, o respeito às escolhas e a promoção de um ambiente de cuidado humanizado, contribui diretamente para a redução dessas complicações, oferecendo um cuidado seguro e baseado em evidências científicas^12^. Entretanto, apesar das evidências favoráveis e dos esforços de implementação e expansão desse modelo assistencial, em 2024, 98% dos nascimentos no país ocorreram em hospitais, o que evidencia a restrita incorporação dos CPNp na atenção ao parto no Brasil^9^.

A implementação dos CPNp no Brasil está vinculada à Rede Cegonha, recentemente atualizada para Rede Alyne, como uma estratégia de reestruturação do modelo de atenção à saúde materna e infantil no Brasil. Esta, que compõe a Rede de Atenção à Saúde do Sistema Único de Saúde (SUS), visa garantir às mulheres o direito ao planejamento reprodutivo, à atenção humanizada durante a gravidez, ao parto e ao puerpério, e às crianças, o direito ao nascimento seguro e ao desenvolvimento saudável^16^.

Além das diretrizes nacionais^17^, a assistência desenvolvida nos CPNp no Brasil tem consonância com as metas globais de saúde materna estabelecidas pela OMS. A organização defende que o modelo de cuidado nos CPNp, centrado na mulher e conduzido por enfermeiras obstétricas e obstetrizes, tem o potencial de reduzir a morbimortalidade materna e neonatal em contextos de baixo risco^4^. Estudos internacionais corroboram essa abordagem, demonstrando que partos realizados em unidades de cuidados de baixo risco apresentam desfechos igualmente seguros, ou até melhores, quando comparados aos realizados em ambientes hospitalares convencionais, desde que haja infraestrutura adequada e critérios rigorosos para a admissão das gestantes^14,15^. Essa abordagem é reforçada pelas evidências que mostram que os partos assistidos por obstetrizes e enfermeiras obstétricas são associados a menores taxas de intervenções, como cesarianas, episiotomias e uso de ocitocina, além de promover maior satisfação materna e melhores desfechos neonatais^13^.

Apesar das evidências favoráveis, os CPNp mantêm limitada inserção na atenção ao parto no Brasil, e a produção científica local ainda não dispõe de estudos de abrangência nacional que avaliem seus resultados com base em indicadores padronizados. Diante desse cenário, este estudo tem como objetivo analisar os resultados assistenciais relacionados aos indicadores de monitoramento e avaliação dos CPNp em funcionamento no âmbito do SUS do país.

## MÉTODOS

### Tipo de Estudo

O Nascer nas Casas de Parto do Brasil é um estudo transversal, descritivo e analítico, de abrangência nacional, integrado ao Nascer no Brasil II. O protocolo com a metodologia do estudo está disponível em Leister et al.^18^

### Local do Estudo

Fazem parte do Nascer nas Casas de Parto todos os oito CPNp do país que registraram, em 2019, mais de 100 partos no Sistema de Informações sobre Nascidos Vivos e seus hospitais de referência para coleta de dados relativos aos desfechos das transferências intraparto, pós-parto e neonatal. À época da seleção dos CPNp, havia 13 CPNp inseridos no Cadastro Nacional de Estabelecimentos de Saúde, porém somente oito foram elegíveis para o estudo. Os estabelecimentos estão localizados em São Paulo (n = 2), Bahia (n = 1), Distrito Federal (n = 1), Minas Gerais (n = 1), Pará (n = 1), Pernambuco (n = 1) e Rio de Janeiro (n = 1). Dados sobre a seleção dos CPNp e hospitais para o estudo estão disponíveis em Leister et al.^18^

### Participantes do Estudo de Coleta de Dados

A população corresponde a todas as gestantes que foram admitidas em trabalho de parto em um dos CPNp do estudo. Não foram convidadas a participar gestantes que se evadiram da casa de parto ou hospital antes da alta, que foram transferidas ou tiveram seu bebê transferido para outro hospital que não tenha sido o de referência do CPNp, que fizeram a entrega legal do bebê e as que não entendiam o idioma português.

A coleta de dados ocorreu de setembro de 2022 a março de 2025 por equipe de cerca de 40 pessoas, todas enfermeiras ou obstetrizes, por meio de entrevistas e dados de prontuário. No período, 1.631 mulheres foram admitidas nos oito CPNp. Fizeram parte da amostra 1.407 mulheres (Quadro).

**Quadro:** Número de partos em 2019, amostra prevista e coletada no Nascer nas Casas de Parto, Brasil, 2022–2025.

Casa de parto	Partos em 2019	Amostra prevista	Amostra coletada
Casa de parto 1	886	468	471
Casa de parto 2	639	337	177^a^
Casa de parto 3	408	215	215
Casa de parto 4	407	215	216
Casa de parto 5	211	113	115
Casa de parto 6	153	81	81
Casa de parto 7	130	69	70
Casa de parto 8	118	62	62
**TOTAL**	**2.952**	**1.560**	**1.407**

^a^ Fechamento do Centro de Parto Normal peri-hospitalar no decorrer do período de coleta de dados.

### Indicadores de Monitoramento e Avaliação dos CPNp

Foram analisados os indicadores de monitoramento e avaliação dos CPN, conforme estabelecido em Portaria do MS, que normatiza o funcionamento e o acompanhamento desses serviços no âmbito do SUS. Esses indicadores contemplam parâmetros relacionados à admissão das gestantes, condução do trabalho de parto, intervenções realizadas, desfechos maternos e neonatais, bem como taxas de transferência intraparto, pós-parto e neonatal^8^.

### Variáveis do Estudo

#### Exposição: características sociodemográficas e obstétricas

As variáveis foram: Idade em anos (contínua, calculada a partir da data de nascimento); Raça/cor (branca; preta; asiática; parda; indígena); Escolaridade (ensino fundamental incompleto; ensino fundamental completo; ensino médio completo; ensino superior completo ou mais); Trabalho remunerado (não, sim); Situação conjugal (não tem companheiro(a); tem companheiro(a)); Número de gestações (contínua); Número de partos normais anteriores (contínua); Número de cesarianas anteriores (contínua); Idade gestacional no parto – contínua (em semanas); Fatores de risco na gestação atual (descritiva).

## Desfecho

Correspondem a todos os 12 indicadores de monitoramento e avaliação dos CPNp que foram divididos em:

**a.Dados sobre os partos**

Indicador 1:

1a) número de partos atendidos;

1b) porcentagem de partos atendidos por profissional de enfermagem e da medicina;

1c) porcentagem de partos de adolescentes;

1d) porcentagem de partos na água;

1e) porcentagem de partos em posição vertical.

**b.Dados sobre o períneo**

Indicador 2: porcentagem de episiotomia.

Indicador 3: porcentagem de lacerações de primeiro e segundo graus.

Indicador 4: porcentagem de lacerações de terceiro e quarto graus.

**c.Dados sobre intervenções**

Indicador 5: porcentagem de amniotomia/rotura artificial de membrana.

Indicador 6: porcentagem de parto com ocitocina no segundo estágio do parto.

**d.Dados sobre acompanhante**

Indicador 7: porcentagem de mulheres com acompanhante durante o trabalho de parto, parto e puerpério.

**e.Dados sobre o acompanhamento pré-natal**

Indicador 8: porcentagem de mulheres atendidas com seis ou mais consultas de pré-natal.

**f.Dados sobre o recém-nascido (RN):**

Indicador 9:

9a) Porcentagem de RN com peso ao nascer menor de 2.500 g ou maior de 4.000 g ;

9b) Porcentagem de RN com idade gestacional menor que 37 semanas e maior que 41 semanas;

9c) Porcentagem de RN com Apgar menor que sete no quinto minuto de vida;

9d) Porcentagem de RN com contato pele a pele ininterrupto e imediato após o nascimento.

**g.Dados sobre as transferências**

Indicador 10: porcentagem de transferência intraparto, discriminada por motivo da transferência.

Indicador 11:

11a) Porcentagem de transferência pós-parto, discriminada por motivos da transferência;

11b) Porcentagem de transferência neonatal, discriminada por motivos da transferência.

**h.Dados sobre atendimento médico**

Indicador 12: porcentagem de avaliação/procedimento médico obstétrico ou pediátrico no CPNp (refere-se à proporção de avaliações ou procedimentos realizados por médico obstetra ou pediatra nas dependências do próprio CPNp).

**i.Outros dados relevantes: tipo de parto e manobra de Kristeller.**

## Análise dos Dados

Os dados foram analisados por meio do programa estatístico R (*R Foundation for Statistical Computing*) de forma descritiva, com valores absolutos e relativos para cada desfecho.

## Aspectos Éticos

O estudo foi aprovado pelos Comitês de Ética em Pesquisa da Escola Nacional de Saúde Pública Sérgio Arouca da Fundação Oswaldo Cruz (parecer nº 5.192.349); Fundação de Ensino e Pesquisa em Ciências da Saúde (parecer nº 5.243.029); Hospital Sofia Feldman/Fundação de Assistência Integral à Saúde (parecer nº 5.256.617); Secretaria Municipal de Saúde do Rio de Janeiro (parecer nº 5.218.319) ; Secretaria Municipal da Saúde de São Paulo (parecer nº 5.251.421). Ao serem convidadas para a pesquisa, as participantes assinaram o Termo de Consentimento Livre e Esclarecido, autorizando a realização da entrevista e coleta de dados dos prontuários.

## RESULTADOS

No período de setembro de 2022 a março de 2025, 1.631 mulheres foram admitidas nos oito CPNp. Das 1.631 mulheres admitidas nos CPNp, 62 (3,8%) não atendiam aos critérios de elegibilidade. Entre as 1.569 elegíveis, 52 (3,3%) recusaram-se a participar do estudo. Ainda, houve 110 (7,0%) perdas, devido à alta das puérperas antes da abordagem para o recrutamento.

As características socioeconômicas e obstétricas estão apresentadas na Tabela 1. As participantes tinham em média 26,5 anos e pouco mais da metade se autodeclarou parda (52,4%). A maioria tinha ensino médio completo (57,0%), parceria (87,8%) e trabalhava (55,5%). Quanto aos dados obstétricos, a maioria era primigesta, 44,2% com parto vaginal anterior e apenas 2,4% com cesariana anterior (ambos os dados não mostrados em tabela). A grande maioria pariu com gestação a termo (96,9%).

**Tabela 1:** Características socioeconômicas e obstétricas das participantes do Nascer nas Casas de Parto do Brasil. São Paulo, 2022–2024.

Variável	n ou média (DP)	% ou número máximo–mínimo
Idade (n = 1.407)	26,5 (5,8)	13–46
10 a 19 anos	147	10,4
20 a 34 anos	1.117	79,4
≥ 35 anos	143	10,2
Raça/cor (n =1.407)		
Branca	358	25,4
Preta	295	21
Asiática	8	0,6
Parda	737	52,4
Indígena	9	0,6
Escolaridade (n = 1.405)		
Ensino fundamental incompleto	94	6,7
Ensino fundamental completo	202	14,4
Ensino médio completo	801	57,0
Ensino superior completo	308	21,9
Trabalho remunerado (n = 1.406)		
Sim	781	55,5
Não	625	44,5
Situação conjugal (n = 1.405)		
Com parceria	1.233	87,8
Sem parceria	172	12,2
Gestações (n = 1.405)	1,9 (1,1)	1–8
1	782	55,6
2 a 3 (1 a 2 partos anteriores)	556	39,6
4 a 5 (≥ 3)	67	4,8
Partos vaginais anteriores (n = 722)	1,3 (0,9)	0–7
Nenhum	101	14,0
1	412	57,1
2	147	20,4
3	40	5,5
≥ 4	22	3,0
Cesarianas anteriores (n = 722)	0,1 (0,2)	0–2
Nenhuma	685	94,9
1	34	4,7
2	3	0,4
≥ 3	0	0,0
Idade gestacional no parto (n = 1.407)	39,0 (0,7)	36–41
< 37 semanas	1	0,1
37 a 40 semanas	1.324	96,9
41 semanas	65	4,7

DP: desvio-padrão.

A Tabela 2 apresenta os resultados dos indicadores 1 a 6, e 12 da Portaria do MS. Estes são relativos ao atendimento ao parto, desfecho perineal, intervenções e atendimento médico. Todos os indicadores se referem a todos os partos de mulheres admitidas para parto nos CPNp, independentemente de onde pariram, se nas casas de parto ou nos hospitais de referência após transferência intraparto. A taxa de parto normal entre as participantes foi de 92,0%, seguida pela cesariana (6,2%).

**Tabela 2:** Indicadores 1 a 6 e 12 da Portaria no 11/2015 do Ministério da Saúde. Nascer nas Casas de Parto do Brasil, 2022–2025.

Indicador	n	%
Indicador 1a): número de partos atendidos	1.407	100
Tipo de parto		
Normal	1.294	92,0
Cesariana	87	6,2
Fórceps/vácuo-extrator	26	1,8
Indicador 1b): partos atendidos por profissional de enfermagem e da medicina (n = 1.406)^a^		
Enfermeira obstetra/obstetriz	1.202	85,5
Medicina (médico obstetra)	204	15,5
Indicador 1c): partos de adolescentes (< 20 anos)	147	10,4
Indicador 1d): partos na água^b^ (n = 1.294)	162	12,5
Indicador 1e): posição para o parto^b^ (n = 1.294)		
Posição litotômica dorsal	16	1,2
Deitada de costas sem perneira	30	2,3
Posições não litotômicas dorsal		
Sentada^c^	425	32,7
Semissentada	374	28,8
Quatro apoios	176	13,6
Lateral	123	9,5
Cócoras^c^	103	8,0
Em pé^c^	41	3,7
Outra posição	3	0,2
Indicador 2: episiotomia^b^ (n = 1.320)	29	2,2
Períneo íntegro	381	28,8
Laceração sem indicação de grau	27	2,0
Indicador 3: lacerações de primeiro e segundo graus^b^ (n = 1.320)	855	64,8
Lacerações de primeiro grau	457	34,6
Lacerações de segundo grau	398	30,2
Indicador 4: lacerações de terceiro e quarto graus^b^ (n = 1.320)	28	2,1
Lacerações de terceiro grau	25	1,9
Lacerações de quarto grau	3	0,2
Indicador 5: amniotomia/rotura artificial de membranas (n = 1.407)	310	22,0
Indicador 6: parto com ocitocina no segundo estágio (n = 1.407)	178	12,7
Manobra de Kristeller (n = 1.407)	13	0,9

^a^ Contabilizados somente os prontuários e as entrevistas que continham informações sobre o profissional que atendeu ao parto.

^b^ Excluíram-se as cesarianas, fórceps e vácuo.

^c^ Consideradas posições verticais.

A maioria dos partos foi atendida por profissionais da enfermagem (85,5%), ou seja, obstetrizes, enfermeiras obstétricas, enfermeiras sem especialização ou residentes de enfermagem obstétrica. Adolescentes representaram 10,4% das participantes. A taxa de parto na água foi de 12,5%, e as posições sentada (32,7%) e semissentada (28,8%) foram as mais adotadas pelas parturientes. A episiotomia foi feita em 2,2% das participantes e as lacerações de terceiro e quarto graus ocorreram em 2,1% delas. A amniotomia foi praticada em 22,0% dos partos e a ocitocina administrada em 12,7%. A manobra de Kristeller, que não faz parte dos indicadores, mas foi adicionada à Tabela 2, foi realizada em 0,9% dos partos.

A Tabela 3 apresenta dados relativos aos indicadores 7, 8 e 9 da Portaria n^o^ 11/2015 do MS, que são: a presença de acompanhante durante toda a internação, o número de consultas pré-natais e os dados sobre o nascimento dos bebês. Da internação à alta, 97,4% das mulheres tiveram a presença ininterrupta de acompanhante. A menor porcentagem de presença de acompanhante foi durante o procedimento de analgesia (81,5%). A maioria realizou seis ou mais consultas de pré-natal (94,5%), 5,2% dos bebês nasceram com peso menor de 2.500 g ou maior de 4.000 g, idade gestacional menor de 37 semanas ou maior de 41 semanas representou 4,7% dos nascimentos. Somente 0,4% dos recém-nascidos tiveram Apgar menor que sete no quinto minuto de vida e a maioria teve contato pele a pele ininterrupto por uma hora após o nascimento (93,3%).

**Tabela 3:** Indicadores 7 a 9 da Portaria no 11/2015 do Ministério da Saúde. Nascer nas Casas de Parto do Brasil, 2022–2025.

Indicador	n	%
Indicador 7: presença de acompanhante durante o trabalho de parto, parto e puerpério (n = 1.390)	1.381^a^	97,4
Acompanhante durante a admissão	1.386	99,7
Acompanhante durante o trabalho de parto	1.386	99,7
Acompanhante durante a analgesia (n = 227^b^)	185	81,5
Acompanhante durante o parto	1.382	99,4
Acompanhante durante o puerpério	1.390	100
Indicador 8: pré-natal realizado em seis ou mais consultas de pré-natal (n = 1.368)	1.293	94,5
Indicador 9a): peso do recém-nascido < 2.500 g e > 4.000 g (n = 1.393)	73	5,2
Peso < 2.500 g	27	1,9
Peso > 4.000 g	46	3,3
Indicador 9b): idade gestacional do recém-nascido < 37 semanas e > 41 semanas (n = 1.390)	66	4,7
Idade gestacional < 37 semanas	1	0,1
Idade gestacional > 41 semanas	65	4,6
Indicador 9c): Apgar do recém-nascido < 7 no quinto minuto (n = 1.401)	6	0,4
Indicador 9d): recém-nascido com contato pele a pele ininterrupto e imediato após o nascimento (n = 1.374)	1.282	93,3

^a^ Para este cálculo não se levou em consideração a presença de acompanhante durante a analgesia.

^b^ Número de partos com analgesia.

A Tabela 4 apresenta os indicadores 10 e 11 sobre as transferências intraparto, pós-parto materna e neonatal com seus respectivos motivos. A maior frequência de transferências intraparto ocorreu por motivos obstétricos, sendo os principais a parada de progressão (4,6%) e a alteração dos batimentos cardíacos fetais (4,2%). Em seguida, destacou-se como motivo de transferência a solicitação da parturiente para possível analgesia hospitalar (4,2%). Em relação à transferência materna pós-parto, motivos clínicos e obstétricos foram prevalentes, sendo a maioria por retenção placentária (0,5%), pico hipertensivo (0,3%) e hemorragia pós-parto (0,3%). Na transferência neonatal, os principais motivos clínicos foram desconforto respiratório (0,9%) e icterícia precoce (0,8%).

**Tabela 4:** Indicadores 10 a 11b da Portaria no 11/2015 do Ministério da Saúde. Nascer nas Casas de Parto do Brasil, 2022–2025.

Indicador	n	%
**Indicador 10: transferência intraparto**	**272**	**19,3**
Motivos obstétricos	190	13,5
Parada de progressão (definida por protocolo institucional)	64	4,6
Alteração dos batimentos cardíacos fetais	59	4,2
Líquido meconial	39	2,8
Bolsa rota prolongada (definida por protocolo institucional)	26	1,8
Apresentação anômala	1	0,1
Taquissitolia	1	0,1
Motivos clínicos	20	1,4
Pico hipertensivo ou alterações de sinais vitais	14	1,0
Alteração de exames laboratoriais	4	0,3
Crise asmática	1	0,1
Crise de ansiedade	1	0,1
Motivos pessoais / profissionais / institucionais	62	4,4
Solicitação materna	59	4,2
Plantão sem profissional clínico disponível	2	0,1
Contato com pessoa covid-19 positivo	1	0,1
**Indicador 11a): transferência pós-parto**	**21**	**1,5**
Motivos obstétricos	11	0,9
Retenção placentária	6	0,5
Laceração perineal de terceiro ou quarto graus	4	0,3
Laceração de colo uterino	1	0,1
Motivos clínicos	10	0,6
Pico hipertensivo	5	0,3
Hemorragia pós-parto	5	0,3
**Indicador 11b): transferência neonatal**	**41**	**2,9**
Motivos obstétricos	1	0,1
Cefalohematoma	1	0,1
Motivos clínicos	39	2,8
Desconforto respiratório	13	0,9
Icterícia precoce	12	0,8
Alterações do ritmo cardíaco	4	0,3
Suspeita de infecção	4	0,3
Suspeita de alterações congênitas	3	0,2
Apneia	1	0,1
Hipoglicemia	1	0,1
Mãe VDRL^a^ positivo	1	0,1
Motivos pessoais/profissionais/institucionais	1	0,1
Contato com pessoa covid-19 positivo	1	0,1

^a^ VDRL (*Venereal Disease Research Laboratory*): exame de sangue para a triagem e acompanhamento da sífilis.

## DISCUSSÃO

Este estudo descreve o cuidado durante o parto e nascimento em oito CPNp brasileiros, com base em indicadores utilizados pelo MS para o monitoramento e a avaliação desses estabelecimentos. A análise dos dados evidencia que a assistência nos CPNp é de qualidade para gestações de baixo risco, em consonância com achados de estudos nacionais e internacionais.

Apesar dos resultados favoráveis, o funcionamento e a expansão dos CPNp ainda são temas controversos. Destacam-se a oferta limitada e a distribuição desigual desses serviços no território nacional, a necessidade de consolidação da integração com a rede hospitalar e as tensões institucionais relacionadas ao modelo assistencial, fatores que dificultam sua consolidação e sustentabilidade a longo prazo^8,12,19^. Ainda assim, em 2024, o governo federal lançou o novo Programa de Aceleração do Crescimento (Novo PAC), que prevê investimentos na construção e modernização de CPNp em todo o país^22^.

Um ponto crucial para a segurança da assistência oferecida em CPNp é a seleção criteriosa de mulheres com baixo risco de complicações. Contudo, não existe consenso mundial sobre a definição precisa de “baixo risco”. Alguns países possuem diretrizes para encaminhamento a um profissional especializado durante o pré-natal, enquanto outros possuem apenas critérios para escolha do local do parto. A existência de diretrizes claras oferece segurança tanto para profissionais quanto para gestantes, orientando a referência a locais seguros para o nascimento. Nesse sentido, destaca-se o Manual Técnico das Casas de Parto do Município de São Paulo^23^.

Ainda há escassez de pesquisas brasileiras robustas que avaliem mortalidade materna e morbidade grave segundo o local do parto, limitando a formulação de políticas públicas com dados consistentes. No entanto, estudos recentes indicam interesse crescente na assistência fora do hospital, liderada por obstetrizes e enfermeiras obstétricas, que apresentam desfechos neonatais semelhantes aos do ambiente hospitalar e com taxas menores de intervenções como episiotomias, cesarianas e uso de alívio farmacológico da dor^15,24,25^.

Este estudo encontrou altas taxas de parto normal (91,9%) entre mulheres de baixo risco que planejaram parto em CPNp, resultado alinhado a estudos recentes no Brasil e no exterior^20,24^. As baixas taxas de intervenções, como episiotomia, amniotomia, uso de ocitocina e manobra de Kristeller, combinadas a altas taxas de imersão em água, presença de acompanhante e adoção de posições não litotômicas, indicam que o cuidado oferecido está alinhado às recomendações da OMS, priorizando a promoção da saúde e um modelo biopsicossocial e humanístico do parto^3,27,28^. Esses achados também sugerem a incorporação de dimensões do cuidado seguro e respeitoso, como suporte contínuo e autonomia, ampliando o conceito de qualidade para além dos desfechos biomédicos^5^.

Esses resultados podem ser explicados tanto pelo perfil das mulheres que escolhem o parto em CPNp quanto pelo modelo de cuidado oferecido, que privilegia o atendimento individualizado (1:1) durante o trabalho de parto. Meta-análises de Bohren et al.^29^ e Sandall et al.^13^ demonstram os benefícios do modelo de continuidade do cuidado e do suporte individualizado, refletidos em maiores taxas de parto vaginal espontâneo, menor uso de intervenções, partos instrumentais e cesáreas, sem prejuízo dos desfechos neonatais, quando comparados a partos de baixo risco em ambiente hospitalar.

As frequências de transferências durante trabalho de parto, pós-parto e puerpério encontradas são semelhantes às relatadas na literatura internacional^24,26,30^. A estratificação dos motivos em causas obstétricas, clínicas e institucionais, ainda pouco explorada em estudos sobre CPNp, amplia a compreensão da dinâmica assistencial desses serviços.

Nos Estados Unidos, o número de pessoas que optam por dar à luz em CPNp dobrou nos últimos 20 anos. A experiência norte-americana destaca a importância da integração dos CPNp ao sistema regional de atenção perinatal, pois isso melhora os desfechos ao estabelecer fluxos definidos para transferências durante o pré-natal e parto, além de favorecer a comunicação eficaz e cooperação contínua entre profissionais^31^.

O direito à informação e à escolha consciente deve ser assegurado às gestantes, para que a decisão sobre o local e o modelo de parto seja informada desde o início da gestação, à luz das preferências individuais e do perfil de risco obstétrico, sendo fundamental que as opções de profissionais e ambientes sejam apresentadas de forma clara e oportuna e que, para mulheres de baixo risco, haja acesso a evidências consistentes sobre os desfechos em CPNp^32,33^.

Nessa perspectiva, este estudo contribui ao adotar análise por intenção de tratar, considerando o planejamento inicial de parto em CPNp, o que permitiu incluir todas as pessoas admitidas, independentemente de transferência, reduzindo vieses de seleção e ampliando a validade interna das estimativas dos desfechos perinatais, inclusive hospitalares. Além disso, oferece dados para sustentar políticas de incentivo tanto ao parto atendido por obstetrizes e enfermeiras obstétricas, quanto para ambientes extra-hospitalares.

## CONCLUSÕES

Nos CPNp analisados, observaram-se baixas taxas de intervenções como amniotomia, uso de ocitocina, manobra de Kristeller e parto em posição litotômica, associadas a elevadas proporções de boas práticas, como presença de acompanhante, parto na água e contato pele a pele ininterrupto, configurando um modelo assistencial alinhado às recomendações contemporâneas para o cuidado ao parto de baixo risco. Entre as mulheres com parto planejado nesses serviços, as reduzidas taxas de cesariana (6,2%), parto vaginal instrumental (1,8%), lacerações graves (2,1%) e Apgar menor que sete no quinto minuto de vida (0,4%) reforçam a segurança do modelo, enquanto as transferências, predominantemente motivadas por indicações obstétricas e clínicas não emergenciais, relacionadas à necessidade de monitoramento e prevenção de riscos, indicam adequada articulação com a retaguarda hospitalar. Em conjunto, esses resultados evidenciam o potencial dos CPNp para qualificar a atenção perinatal no Brasil, integrando segurança clínica e promoção de experiências positivas e respeitosas no parto.
